# Comparative genomics identifies distinct lineages of *S*. Enteritidis from Queensland, Australia

**DOI:** 10.1371/journal.pone.0191042

**Published:** 2018-01-16

**Authors:** Rikki M. A. Graham, Lester Hiley, Irani U. Rathnayake, Amy V. Jennison

**Affiliations:** Public Health Microbiology, Forensic and Scientific Services, Queensland Department of Health, Coopers Plains, Queensland, Australia; Laboratoire National de Santé, LUXEMBOURG

## Abstract

*Salmonella enterica* is a major cause of gastroenteritis and foodborne illness in Australia where notification rates in the state of Queensland are the highest in the country. *S*. Enteritidis is among the five most common serotypes reported in Queensland and it is a priority for epidemiological surveillance due to concerns regarding its emergence in Australia. Using whole genome sequencing, we have analysed the genomic epidemiology of 217 *S*. Enteritidis isolates from Queensland, and observed that they fall into three distinct clades, which we have differentiated as Clades A, B and C. Phage types and MLST sequence types differed between the clades and comparative genomic analysis has shown that each has a unique profile of prophage and genomic islands. Several of the phage regions present in the *S*. Enteritidis reference strain P125109 were absent in Clades A and C, and these clades also had difference in the presence of pathogenicity islands, containing complete SPI-6 and SPI-19 regions, while P125109 does not. Antimicrobial resistance markers were found in 39 isolates, all but one of which belonged to Clade B. Phylogenetic analysis of the Queensland isolates in the context of 170 international strains showed that Queensland Clade B isolates group together with the previously identified global clade, while the other two clades are distinct and appear largely restricted to Australia. Locally sourced environmental isolates included in this analysis all belonged to Clades A and C, which is consistent with the theory that these clades are a source of locally acquired infection, while Clade B isolates are mostly travel related.

## Introduction

*Salmonella* infection is a leading cause of gastroenteritis, with non-typhoidal *Salmonella enterica* estimated to cause approximately 94 million cases globally per year [1]. In 2015, there were 17 012 notifications of Salmonellosis in Australia, 4811 of which were from the state of Queensland (QLD), an increase of 67% on the 2887 QLD notifications received in 2010, and the highest level of *Salmonella* nofitications in Australia [2, 3]. More than 2500 serotypes of *S*. *enterica* have been identified, however two of these serotypes, *S*. Enteritidis and *S*. Typhimurium account for approximately 60% of cases globally [4]. In Australia, *S*. Enteritidis is among the five most common Salmonella serotypes reported, [5, 6], and although rates of notification are not as high as those for *S*. Typhimurium, concerns regarding its emergence in Australia make it a priority for epidemiological surveillance. The majority of *S*. Enteritidis cases in Australia are associated with travel, and nationally only approximately 10% are thought to be locally acquired [6]. However, in QLD the number of locally acquired infections is the highest in Australia, and the relative proportion of locally acquired infections compared to overseas acquired infections is greater than the national average and that of other states at 20% [6]. Prior to this study, molecular surveillance has not been performed on *S*. Enteritidis cases isolated in Queensland and local sources of infection are unclear.

*S*. Enteritidis is reportedly one of the most genetically homogeneous serotypes of *Salmonella* [7], and while current methods of typing such as Phage typing and MLST are able to classify *S*. Enteritidis isolates into broad groups, they lack the high level of resolution suitable for delineating epidemiologically linked clusters. Whole genome sequencing (WGS) has been successfully used as a tool in epidemiological surveillance and outbreak investigations and it has the ability to provide a very high level of resolution, comparing differences across the entire genome. In order to take advantage of the improved resolution that WGS provides, the Public Health Microbiology laboratory at the QLD Department of Health routinely sequences all contemporary *S*. Enteritidis isolates received by the laboratory. WGS analysis has identified the existence of 3 distinct genomic clades among the QLD *S*. Enteritidis isolates sequenced and we report on comparative genomics to determine how these clades fit within the global *S*. Enteritidis population.

## Methods

### Sequencing of bacterial strains

*Salmonella enterica* serovar Enteritidis strains included in this study were isolated from clinical (n = 206) or environmental samples (n = 11) as outlined in S1 Table. DNA was extracted from isolates grown overnight at 37°C on horse blood agar, using the QiaSymphony DSP DNA Mini kit (Qiagen) according to the manufacturer’s protocol. DNA was prepared for sequencing using the Nextera XT kit (Illumina) and sequenced on the NextSeq500 using the NextSeq 500 Mid Output v2 kit (300 cycles) (Illumina) according to the manufacturer’s instructions. Raw sequence files and associated metadata have been submitted to the European Nucleotide Archive with project accession number PRJEB22598.

### Bioinformatic analysis

Sequences generated were quality trimmed using Trimmomatic v0.36 [8]. Core SNPs were determined by mapping reads to the genome of *S*. Enteritidis P125109 (Genbank accession number NC_011294) using the Snippy pipeline (https://github.com/tseemann/snippy), and identifying SNPs that were present in greater than 90% of reads at a depth of at least 10x. Core SNPs were aligned and used to generate a maximum likelihood tree using the RAxML wrapper in Geneious R10 (Biomatters, New Zealand), using the GTR CAT model and 100 bootstrap replicates [9]. Sequences were de novo assembled into contigs using the SPAdes v3.10.1 assembler [10]. Contigs were ordered to the P125109 genome, and genome alignments performed using Mauve 2.4.0 [11]. MLST was performed in RidomSeqSphere+ 4.1.0 (Ridom, Germany) using the Achtman scheme hosted at Enterobase (https://enterobase.warwick.ac.uk/species/index/senterica) [12]. AMR genes were detected using Abricate with the ResFinder and CARD databases (https://github.com/tseemann/abricate), and chromosomal point mutations associated with antimicrobial resistance were detected using ResFinder [13, 14]. The molecular serotype of the QLD isolates was assessed using SeqSero [15].

## Results and discussion

### Genomic epidemiology

**Fig 1:**
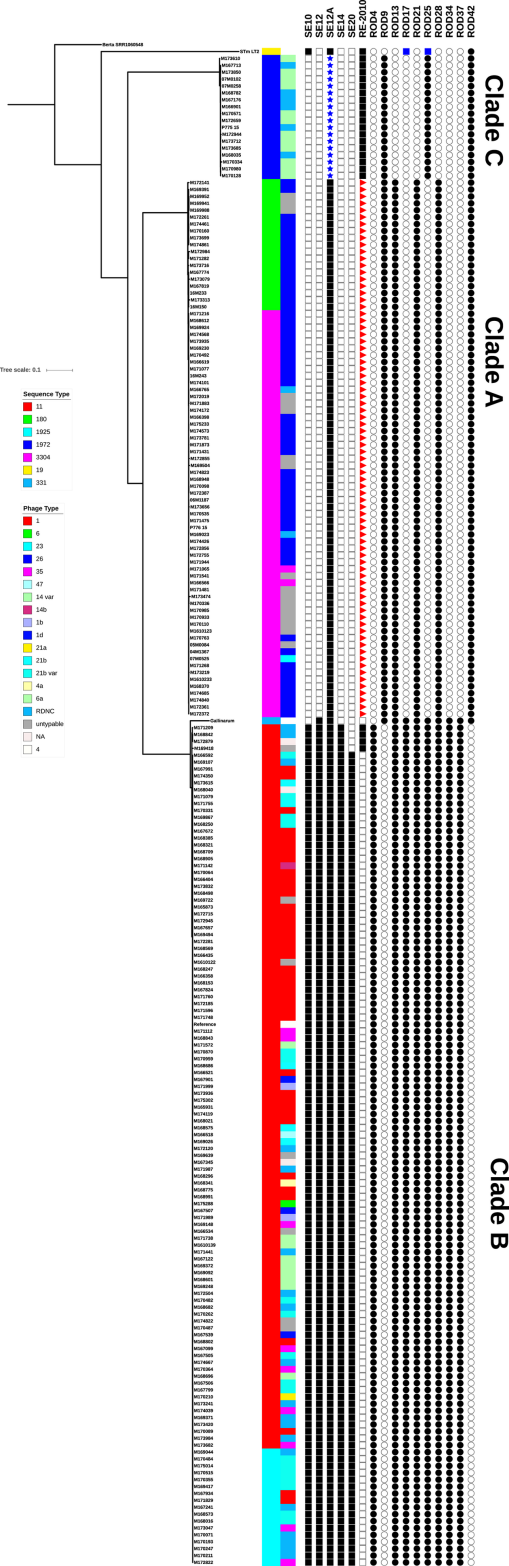
Maximum-likelihood phylogeny of QLD *S*. Enteritidis isolates, *S*. Typhimurium LT2 and *S*. Gallinarum 287/91 rooted to *S*. Berta. Isolates cluster into three clades as indicated. The scale bar shows substitutions per site. Sequence type and Phage type are indicated by colour as shown in the legend. The presence and absence of regions of difference (RODs) and prophages are also indicated in the following order: φSE10, φSE12, φSE12A, φSE14, φSE20, RE-2010, ROD4, ROD9, ROD13, ROD17, ROD21, ROD25, ROD28, ROD34, ROD37, ROD42, with a white box indicating absence and a black box indicating presence. Blue stars, red triangles and blue boxes indicate partial presence of the prophage.

In order to elucidate the genetic relationship between *S*. Enteritidis isolates in the QLD community and how patient isolates relate to those from animals and foods, sequences from 206 clinical and 11 environmental isolates of *S*. Enteritidis (S1 Table), as well as the publicly available reads from one serovar Berta isolate, one *S*. Typhimurium isolate and one *S*. Gallinarum isolate(Genbank accession numbers SRR1060548, NC_003197 and AM933173 respectively), were mapped to the *S*. Enteritidis reference genome P125109 (Genbank accession number NC_011294). Regions identified by BratNextGen [16] as being involved in recombination were excluded from the analysis, and high quality core SNPs were identified using Snippy. In total 43 431 variable sites were identified and an alignment of these SNPs was generated and used to construct a maximum likelihood phylogeny using RAxML with the serovar Berta isolate as an outgroup. The phylogeny showed that the *S*. Enteritidis isolates grouped into 3 distinct clades, which we have named Clades A, B and C (Fig 1). Clade A is approximately 12000 SNPs distant from Clade B and 16000 SNPs distant from Clade C, while the distance from Clade B to Clade C is approximately 17000 SNP*S*. Within each clade there is a relatively low level of diversity with fewer than 500 SNPs found between isolates from the same clade. Clade B had the highest number of isolates with 121 isolates belonging to this clade, while Clade A had 78 and Clade C was the smallest with 18 isolates. Among the isolates tested, 11 were from environmental sources such as food and poultry farms, as detailed in S1 Table. Eight of these clustered with Clade A, and three with Clade C, while none of the environmental isolates belonged to Clade B. There was no association with the environmental source of the isolates and whether it fell into Clade A or C, with isolates from both of these clades being found in chickens and eggs, and isolates from both clades being found in food. Two isolates were isolated from domestic animals, both of which belonged to Clade A (S1 Table).

Isolates from the three clades had distinct phage types (PT) and MLST types (ST). For Clade A isolates, the highest number, 56 (72%) were PT26, 17 (22%) were untypable and the remaining 5 isolates (6%) were made up of PT23, PT35, and reactive but did not conform (RDNC). Clade B isolates showed more variation in phage type with 41 (34%) PT1, 35 (29%) PT35, 19 (16%) RDNC, 18 (15%) PT21b var, 9 (7%) PT6a, 5 (4%) PT21b, with 10 (8%) being untypable or with no phage type information available and the remaining 9 isolates (7%) were made up of PT1b, PT1d, PT6, PT47, PT14b, PT21a, and PT4a. Clade C isolates showed the least phage type variation with 12 (67%) PT14 var and 6 (33%) RDNC (Fig 1). The phage types seen in the QLD isolates are different from those commonly observed in other countries where PT4, PT8 and PT13a are most common [5]. In particular certain phage types are generally found to be predominant in different regions, with PT4 most common in Europe, PT8, PT13 and PT13a most common in North America, and PT1, PT6a and PT21 most common in Asia [17–21]. In the QLD isolates, different phage types were associated with the different clades. Clade B contained high numbers of PT1 and PT6a, PTs commonly seen in Asia, with the next most common PTs in Clade B being PT21b and PT21b var. These are relatively new phage types, with PT21b first described in 1995 in the UK [22]. The presence of globally common PTs in Clade B isolates is consistent with the observation that the majority of these cases were associated with a reported overseas travel history and that the highest number of overseas acquired *S*. Enteritidis infections in Australians is associated with travel to Asia [6]. Clade A largely consists of PT26, a PT not commonly seen in other countries but relatively common among Australian *S*. Enteritidis strains and associated with locally acquired infection [6]. Clade C also consists of an unusual PT, PT14 var as well as a relatively high number of isolates that were reactive but did not conform to known phage types.

The STs seen in the QLD isolates were also associated with the different clades. Clade A isolates were either ST180 (24%) or ST3304 (76%), Clade B were predominately ST11 (86%), but 14% were ST1925 which is a single locus variant of ST11. Clade C isolates were all ST1972 (Fig 1) Among *S*. Enteritidis strains worldwide, ST11 is the most common ST, accounting for 89% of the 17867 *S*. Enteritids entries in the EnteroBase database (http://enterobase.warwick.ac.uk/ - accessed 07/09/2017), and for 95% of *S*. Enteritidis isolated in England and Wales between April 2014 and March 2015 [23]. The single locus variant of ST11, ST1925 had 84 entries in EnteroBase, while ST180, ST3304 and ST1972 have been previously been recorded for a relatively low number of *S*. Enteritidis strains with 19, 2, and 11 entries respectively in the EnteroBase database.

In order to determine how the clades identified in Australian *S*. Enteritidis isolates related to international *S*. Enteritidis strains, the publicly available short read sequence files of 170 *S*. Enteritidis isolates from a range of geographic locations (S2 Table) were downloaded from the European nucleotide archive (ENA) and SNP analysis was performed as described above together with the QLD isolates. A maximum likelihood phylogeny was generated using RaXML with the serovar Berta as an outgroup. As has been described in previous studies [24, 25], we noted the presence of a ‘global epidemic clade’ that includes the reference strain P125109. This clade includes 124 isolates from Asia, Europe and the Americas as well as the 121 Australian Clade B isolates. Deng *et al*., further subdivided this global clade into 5 lineages, with Lineage 3 containing the reference strain P125109 and clinical strains from Thailand and the USA, and Lineage 5 containing clinical and environmental strains from the USA [26]. The clade B strains included in the current study were also observed to divide into these lineages, with the majority of QLD Clade B isolates (n = 117) clustering together with Lineage 3 isolates, into what we have referred to as the P125109-like lineage because it contains the reference strain. The majority of the 63 international strains in this lineage are from Asia, with 33 of the 63 Asian strains included in this analysis clustering into this lineage (S1 Fig), while the remaining 17 strains were from other global locations. Three of the remaining four QLD Clade B isolates clustered into the lineage corresponding with Lineage 5 in Deng *et al*. This lineage contains the LK5 strain and so we have referred to it as LK5-like. This lineage contained strains from a more diverse range of global locations, with many from the USA, Canada and Europe and eight Asian strains. We also observed the presence of a third, smaller subclade consisting entirely of thirteen Asian isolates and one QLD Clade B isolate.

With the exception of a small number of international strains, Clades A and C were almost exclusively comprised of Australian isolates. Clade A also contained two isolates from Germany, one from the UK, one from Vietnam, one from the USA, one from Vanuatu and one from New Zealand (S2 Table). These were all ST 180, one of the STs associated with the QLD Clade A isolates, and the New Zealand strain had previously been described as being a divergent strain of *S*. Enteritidis by Deng *et al*.[26]. In addition to the Australian isolates, Clade C also contained two isolates from the UK and two from the USA (S1 Fig), all of which were also ST1972. The novel African lineages identified by Feasy *et al*., [24] showed a correlation between clade and invasive disease. Unfortunately due to the limited number of invasive *S*. Enteritidis isolated in the time period covered by this study (n = 6) no correlation between invasive disease and clade could be observed for the QLD isolates (S1 Table).

### Comparative genomic analysis

In order to identify similarities and differences between the three clades identified in QLD *S*. Enteritidis, the genomes of isolates from each of the clades were compared to each other and to that of P125109. *De novo* assembled contigs from each isolate were ordered against the P125109 sequence using Mauve and then concatenated. These were then aligned to the P125109 genome using the Mauve plugin in Geneious R10 and regions of difference (RODs) were identified. Specific regions of difference such as prophages, pathogenicity islands and gene sets that have been previously described in studies of the *S*. Enteritidis genome [17, 25, 27, 28] were also investigated using Ridom SeqSphere+. Overall, the genomes of QLD clade B isolates were highly similar to P125109, containing all of the gene sets highlighted as RODs by Thomson *et al* [28], as well as all previously identified pathogenicity islands and with a very similar prophage profile. Clades A and C however, had a number of differences in their genomes when compared to Clade B and P125109 as described below and summarised in Fig 1.

#### Regions of difference

Thomson *et al*., identified 17 gene sets that differed between *S*. Enteritidis, *S*. Gallinarum and *S*. Typhimurium and labelled these as RODs [28]. These RODs contain genes involved in a variety of functions including metabolism, membrane transport and potential virulence factors [28, 29]. Of the 17 RODs, five were absent in Clade A isolates, ROD4, ROD17, ROD25, ROD34, ROD37, while in Clade C isolates, with the exception of ROD25, the same regions were missing as well as ROD13 and ROD28 (Fig 1). A few Clade C isolates also lacked ROD21 but in all the other Clade C isolates there was a different ROD21 which had a maximum identity of 96% and coverage of 43% with ROD21 in Clade A and B isolates. ROD21 in P125109 is a 26.5 kb genomic island which contains genes involved in virulence, and for the global transcriptional silencer H-NS and its antagonist [27]. The ROD21 in Clade C isolates is a 21.3 kb genomic island which is located just downstream of the integration site for P125109 ROD21 and integrates into the tRNA-Asn present in P125109 at SEN_t035. The Clade C ROD21 has an integrase that shares 97% amino acid identity with that from P125109 ROD21 and shares a nucleotide identity above 98% with 8 of the 25 ROD21 genes in P125109 (SEN_RS10280, SEN_RS10310, SEN_RS10315, SEN_RS10335, SEN_RS10400, SEN_RS10405, SEN_RS10410, SEN_RS10415.) ROD21 was found to be absent in *S*. Enteritidis belonging to the East and West African clades reported in [24]. ROD9, which contains genes involved in T6SS is present in a degenerate form in P125109 and in Clade B isolates, but was found to be complete in Clade A and Clade C isolates and is highly similar to the same region in *S*. Gallinarum 297/91 where it includes SPI-19 [30]. ROD42 was another region that was absent in P125109 and Clade B, but present in Clade A and Clade C isolates. ROD42 is present in *S*. Typhimurium LT2 and *S*. Gallinarum 297/91 where it encodes for C4-dicarboxylate transporters [28]. Interestingly, with the exception of ROD42, all of the RODs that were absent in either Clade A or Clade C isolates were also either degenerate or completely absent in *S*. Typhimurium LT2 and are thought to have either been lost by this serovar when it diverged from the common ancestor it shares with *S*. Enteritidis, or to have been acquired independently by *S*. Enteritidis following divergence [28]. The international isolates that clustered into QLD Clades A and C shared the same ROD profiles as the QLD strains from the same clade, with the exception of ROD21, which was absent from 5 of the 7 international Clade A isolates, but present in the two isolates from Germany.

#### Phage content

Prophages are important drivers of diversity in *S*. *enterica*, and there is a high degree of diversity in the prophage content of different serovars of *Salmonella*, and even within serovars. For example, while *S*. Enteritidis P125109 and *S*. Enteritidis LK5 both contain five prophage regions, the actual prophages present in these two strains differ [28, 31]. Likewise, the three clades observed in QLD isolates also demonstrated diversity in their prophage content. Clade B isolates possessed 2 different phage profiles, with some having the same prophage profile as P125109 and some having the same profile as LK5. This difference correlated with the clustering of Clade B isolates with the P125109 lineage or the LK5 lineage in the phylogenetic analysis in S1 Fig. Those in the P125109 lineage contained φSE10, φSE12, φSE12A and φSE14 and φSE20, while those in the LK5 lineage lacked φSE20 but contained the RE-2010 sequence described by Zheng *et al*., located in the same region as in LK5 [25].

The prophage profiles of Isolates belonging to Clade A and Clade C differed from that of the Clade B isolates. In both Clade A and Clade C isolates the integration site for φSE10 was empty. Prophage φSE10 contains the virulence-contributing genes *sseI*, *gtgE*, and *gtgF* and is also absent in serovar Gallinarum [17, 28, 31]. The integration site for φSE14 was also empty for isolates from both Clade A and Clade C. When the *de novo* assembled contigs for each isolate were submitted to SeqSero for molecular serotyping, a region that is commonly used as a marker for *S*. Enteritidis, *sdfI* [26, 32] was found to be absent in isolates from Clade A and Clade C, meaning that they were not identified as *S*. Enteritidis [15]. Investigation of the location of this region indicated that it is found on φSE14. It has previously been reported that φSE14 is unstable and can be spontaneously excised from the chromosome [33], so it is possible that it has been lost from Clade A and Clade C strains. Alternatively, it may have been later acquired by Clade B strains as Porwollik *et al*., found that 2 out of 8 strains isolated prior to 1950 were lacking φSE14 [17]. Virulence studies found that the absence of this prophage had no effect on the ability of *S*. Enteritidis to colonise a mouse model compared to strains that carry φSE14 [33].

In Clade C isolates φSE12 had been replaced by a Gifsy-2 like prophage about 35.3 kb in size located at the same site. The integrase is the same as in φSE12 and there is a high level of identity for five sections of the φSE12 sequence with insertions in between. The five sections are in the same order in both φSE12 and the Gifsy-2. Therefore it appears that the φSE12 sequence in P125109 and other Clade B genotypes is a degenerate form of the Gifsy-2 in Clade C isolates. The φSE12A just downstream of the Gifsy-2 prophage has high identity to the same phage remnant in P125109 except for a missing 1.8 kb sequence corresponding to genes 9–11 in the P125109 prophage. The same sequence is also missing in the corresponding sequence in *S*.Typhimurium LT2 (GenBank NC_003197 nt 1962481–1967919) so that Clade C isolates have virtually the same sequence as LT2. One of the Gifsy-2 genes present in Clades B and C, SEN1140, is reported to be important in early colonisation and inflammation [29].

The situation is somewhat different in Clade A isolates. All Clade A isolates lack all or most of the φSE12 prophage. Some are missing the entire sequence including the integrase, others have a Gifsy-1 prophage of about 51 kb with almost the same integrase as φSE12 located in the φSE12 site and contain a number of φSE12 genes, including the virulence factors *sopE* and *sodC* [28, 31]. Some Clade A isolates have a different Gifsy-1 prophage in the same location as Gifsy-3 in *S*.Typhimurium 14028S (GenBank NC_016856 nt 1284474–1284687). In these strains the φSE12 site is occupied by the integrase and subsequent excisionase from φSE12 followed by the next three genes from the Gifsy-1 found in Clade A strains that carry the Gifsy-1 suggesting an earlier loss of the Gifsy-1 prophage. In all three types of Clade A isolates there is a full φSE12A sequence just downstream of the φSE12 site with high identity to the sequence in P125109.

Isolates from both Clade A and Clade C lack φSE20, which is a 41 kb phage related to φST64B from *S*. Typhimurium [27, 28]. φSE20 is intact in the P125109-like Clade B isolates and in P125109 where it is thought to be a recent acquisition and has been implicated in *S*. Enteritidis virulence in mice and the invasion of chicken ova [34]. Betancor *et al*., have suggested that the presence of φSE20 in *S*. Enteritidis is associated with the emergence of particular isolates as epidemic strains in Uruguay as they have found that 3 out of 6 pre-epidemic isolates lacked φSE20 while only 5 out of 108 epidemic and post-epidemic isolates lacked φSE20 [27]. Similarly, φSE20 was absent in isolates from the 1940s and 1950s tested by Porwollik *et al*., but was present in the more recent isolates [17]. As mentioned above, Clade B LK5-like isolates do not contain φSE20 but do possess a Fels2-like prophage called RE-2010.

When compared to the P125109 genome, Clade C isolates also appear to have a ~42 kb region inserted downstream of SPI-9, between SEN2612 and SEN2613. This sequence is similar to the RE-2010/Fels-2 prophage located in the same region in the LK5 strain and in *S*. Typhimurium LT2 (NC 003197, STM2694), although the prophage does not have all of the Fels2 genes present. Clade A isolates have a ~12 kb region inserted in the same location, also showing similarity to the RE-2010/Fels-2 prophage, however many of the genes are absent.

The international isolates that clustered into QLD Clades A and C shared the same phage profiles as the QLD strains from the same clade.

#### Pathogenicity islands

Salmonella pathogenicity islands (SPI) are clusters of genes that play a role in the pathogenesis of *Salmonella enterica* infections. So far, 21 SPIs have been described [30, 35–40]. The *S*. Enteritidis strain P125109 has 12 SPIs, SPI-1-6, SPI-10, SPI-12, SPI-13, SPI-14, SPI-16, SPI-17 and SPI-19 [28, 41]. The majority of the P125109 SPIs are present in all three QLD clades, SPI-1 to SPI-5 and SPI-9, SPI-10, SPI-13, SPI-14 and SPI-16 are all present and complete in all three clades, although there are some amino acid changes in the coding regions in the Clade A and Clade C isolates.

SPI-6 encodes a T6SS, contains a fimbrial gene cluster and the invasin *pagN* [24, 42]. In P125109 SPI-6 is degenerate with several of the Type VI secretion system genes that are present in *S*. Typhimurium LT2 missing. As with P125109, clade B isolates also have a degenerate SPI-6, however SPI-6 in clade A and clade C isolates is complete and similar to that for *S*. Typhimurium LT2, although with some differences in genes 25–30 of the island in Clade A and genes 27–30 in Clade C.

SPI-17 is a SPI with an undefined function. It is a prophage remnant and contains genes with high homology to P22 phage conversion genes, and contains genes know to be involved in O-antigen conversion in other bacteria [28, 42]. This SPI was found to be present in all Clade B and Clade C isolates, but is absent from isolates belonging to Clade A.

SPI-19 contains genes that encode for a Type VI secretion system. This SPI is largely absent from P125109 which has a 24 kb internal deletion in SPI-19, corresponding with ROD9 described above, with only 16 of the 30 SPI-19 ORFs present [28, 30]. SPI-19 is also degenerate in Clade B isolates, but like ROD9 is present and complete in the Clade A and C isolates.

#### Antimicrobial resistance genes

**Fig 2:**
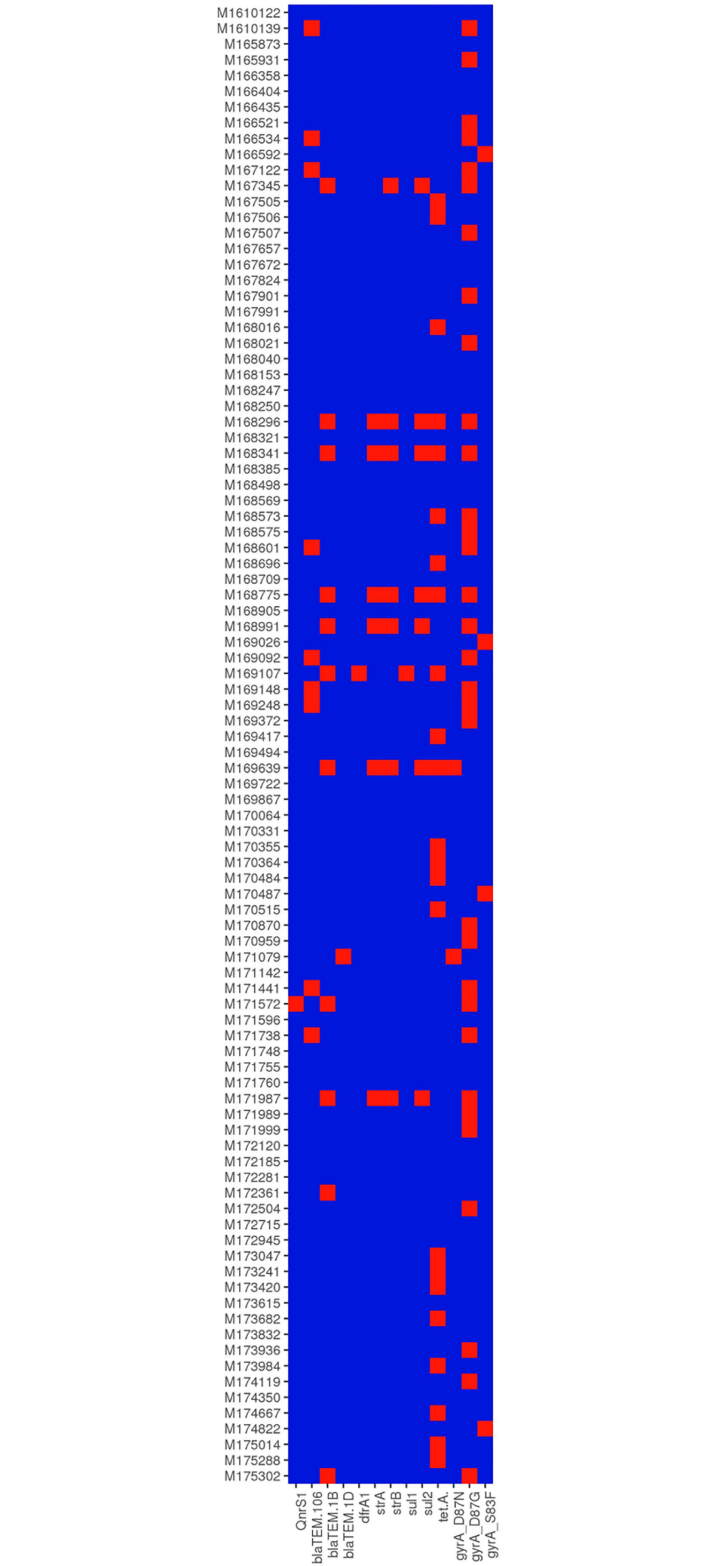
Heatmap showing the presence and absence of acquired antimicrobial resistance genes and point mutations in *gyrA*. Blue boxes indicate absence and red boxes indicate presence of genes or point mutations.

*De novo* assembled contigs were screened for the presence of acquired antimicrobial resistance (AMR) genes using Abricate and defined chromosomal point mutations using ResFinder. As has been previously reported for *S*. Enteritidis, all isolates contained the cryptic aminoglycoside acetyltransferase gene aac(6’)-Iy [24, 43]. Additional acquired AMR genes were detected in 39 (18%) isolates, all of which belonged to Clade B, with the exception of one Clade A isolate that had *bla*_TEM-1B_ present (Fig 2) One isolate contained a marker for quinolone resistance *qnrS1*, which has also been seen in Malaysian *S*. Enteritidis isolates [44]. The tetracycline resistance marker *tet*(A) was the most common antimicrobial resistance gene present, found in 24 isolates, and 21 isolates had a *bla*_TEM_ gene, a gene commonly associated with resistance to extended spectrum β-lactamases in *Salmonella* [45, 46]. One isolate had a marker for trimethoprim resistance, *dfrA1*. This isolate had multiple resistance markers, also carrying *sul1*, *bla*_TEM_ and *tet*(A) genes. Six isolates had the AMR genes *strA* and *strB* as well as *sul2*, *bla*_TEM_ and *tet*(A). Point mutations in the *gyrA* gene, associated with reduced susceptibility to quinolones were found in 38 isolates, all of which belonged to Clade B. Nine isolates had more than one acquired resistance marker, and seven isolates had four or more acquired resistance genes (Fig 2), a finding that is consistent with the incidence of multi-drug resistant *S*. *enterica* reported in other studies [24, 45–48]. This incidence of AMR genes in *S*. Enteritidis is similar to that seen in the global epidemic clade described by Feasy *et al*., [24], however, the almost complete lack of AMR genes in the Clade A and Clade C isolates is somewhat unusual.

#### Plasmid content

Plasmids are also known to play an important role in Salmonella pathogenicity. De novo assembled contigs were searched for the presence of plasmid sequences using PlasmidFinder, and results were confirmed by BLAST [49]. Of the plasmid sequences found, nearly all were found in Clade B isolates. Out of 121 Clade B isolates, 109 had an IncF1B plasmid almost identical to the plasmid from *S*. Enteritidis str. CDC_2010K_0968 (Genbank accession number CP007529). This is a truncated relative of the pSLT plasmid in *S*. Typhimurium str. LT2 (Genbank accession number NC_003277). None of these plasmids contained genes associated with antimicrobial resistance. Forty-six Clade B isolates contained an IncX1 plasmid and nearly all of the acquired antimicrobial resistance genes found in Clade B isolates were associated with these Inc X1 plasmids. There were six related but different IncX1 plasmids found with five different antimicrobial resistance gene profiles. The IncX1 plasmid found in 19 isolates had only tetracycline resistance genes. There were two variants of an IncX1 plasmid in seven isolates with resistance genes bla-_TEM_, streptomycin A and B, sulphonamide 2 and tetracycline A and R. Another nine isolates had an IncX1 with bla-_TEM_ only, and there were three other IncX1 plasmids in singleton isolates, one with bla-_TEM_ only, one with bla-_TEM_, trimethoprim, sulphonamide 1 and tetracycline genes and one with bla-_TEM_ and tetracycline genes. Eight other isolates had IncX1 plasmids with no antimicrobial resistance genes. Two Clade B isolates had related but different IncI1 plasmids.

Among Clade A isolates there were two isolates with the same IncF1 plasmid, which is only distantly related to pSLT and another two isolates had two related but different IncI1 plasmids, one in combination with a colpVC-related plasmid. None of the Clade C isolates had any plasmid or antibiotic resistance genes.

## Conclusions

Many studies of *S*. Enteritidis have commented on the homogeneity of this serovar [7, 26, 50, 51], however it has recently been demonstrated that there are diverse lineages of *S*. Enteritidis that exist globally [24, 41]. We have identified the existence of two additional lineages of *S*. Enteritidis circulating in the QLD population in Australia. These appear to be largely restricted to Australia with strains belonging to these novel clades demonstrating PTs and STs that are uncommon among reported global *S*. Enteritidis strains, including one ST, ST3304 which has only been reported in Australian strains. Previous studies have compared the genome of *S*. Enteritidis with that of different serovars and different phage types and identified differences in prophage content, pathogenicity islands and the presence or absence of genes [17, 25, 27, 28]. By conducting comparative genomic analysis of the QLD isolates compared to *S*. Enteritidis P125109 we have shown that each clade has a unique collection of prophage and genomic islands. Feasy *et al*., proposed that the differences seen in the novel East and West African lineages could indicate different ecological niches outside the human host [24]. This could also be true of the novel Australian lineages, and further investigation of environmental niches of these clades is warranted.

When the QLD isolates were analysed in the context of international strains, it was clear that Clades A and C were highly diverged from the majority of the international strains tested, and that these Clades were comprised mostly of Australian isolates. Strains belonging to Clade A and Clade C do not appear to be exclusive to Australia however, having been isolated and sequenced in other countries (S2 Table). Of the four divergent *S*. Enteritidis described by Deng *et al*., in their study, one from New Zealand (77–0915) clustered with the QLD Clade A strains and another, SARB19 from Switzerland clustered close to Clade C [26]. Despite the existence of strains similar to Clade A and Clade C isolates outside of Australia, it appears that strains belonging to these clades are more prevalent in Australia than they are in other countries and make up a higher proportion of *S*. Enteritidis isolated from cases of Salmonellosis than is seen elsewhere in the world. The number of environmental isolates in this study is small and so the conclusions that can be drawn on environmental sources of QLD *S*. Enteritidis are limited. However, the fact that environmental isolates sourced from QLD were from Clades A and C aligns with the theory that these clades are a source of locally acquired infection, while Clade B isolates are mostly overseas acquired, although we cannot exclude the possibility that Clade B isolates are present in the QLD environment. The isolation of *S*. Enteritidis in swabs from a chicken farm and in egg pulp is not surprising given the knowledge that poultry and poultry products are a source of *S*. Enteritidis, however, it appears that the strains of *S*. Enteritidis associated with chicken in QLD are substantially different from those found in flocks from other geographic locations. In particular the lack of φSE20, proposed to be important for virulence and invasion of the oviduct in chickens [34], in Clade A and C isolates may explain in part the observation that *S*. Enteritidis has not caused the same problems in the Australian poultry industry as has been seen elsewhere in the world. The isolation of two Clade A isolates from domestic animals, may also indicate pets as a potential reservoir for infection. The low presence of AMR genes in the Clade A and Clade C isolates is notable. Australia has strong regulations in place regarding the use of antimicrobials in agriculture and animal processing and *Salmonella enterica* multi drug resistance rates from Australian livestock have been reported as low [52]. It is possible that this absence of resistance markers in locally acquired Clade A and C cases may reflect the absence of significant antimicrobial pressure in as yet unknown local environmental sources.

The bulk of QLD Clade B isolates were found to group into a cluster corresponding with Lineage 3 described by Deng *et al*., [26]. In our analysis, this group consists primarily of Asian strains, and also contains the majority of Asian strains included in this study. The remaining Clade B isolates either clustered into a group corresponding with Lineage 5 described by Deng *et al*., or into a smaller clade consisting entirely of Asian strains. As mentioned above, the PT and ST profiles seen in the QLD Clade B isolates is consistent with them being related to travel, and the clustering of these strains primarily with strains from Asia is consistent with the popularity of travel to Asia among Australians.

Several studies have speculated on the evolution of *S*. Enteritidis and similar serovars [25, 28, 31, 41]. Thomson *et al*., proposed that *S*. Enteritidis PT4 gained φSE12 after diverging from the common ancestor it shares with *S*. Typhimurium. Following this, *S*. Enteritidis then diverged from *S*. Gallinarum and gained φSE14 and φSE20. QLD Clade A and Clade C isolates lack φSE10, φSE14 and φSE20 so it is tempting to speculate that these clades are older than Clade B and that Clade B diverged from these clades before gaining φSE10, φSE14 and φSE20. Clades A and C also possess complete forms of SPI-6 and SPI-17, while in Clade B these genomic islands have degenerated, so it is possible that these genomic islands have degenerated in Clade B following divergence. Other studies have also shown that older *S*. Enteritidis isolates lack φSE14 and φSE20 [17, 27]. Therefore it is possible that Clades A and C represent an older population of *S*. Enteritidis, that has not undergone the same changes seen in Clade B and the global epidemic strains of *S*. Enteritidis. Further study of the evolution of these clades is required before conclusions can be drawn, however such studies would prove beneficial in gaining a greater understanding of the *S*. Enteritidis population in QLD.

## Supporting information

S1 Table QLD *S*. Enteritidis strains included in this study. (XLS)

S2 Table International *S*. Enteritidis strains included in this study. (XLS)

S1 Fig Maximum-likelihood phylogeny of QLD *S*. Enteritidis and international strains, rooted to *S*. Berta. Continent of isolation is indicated by colour as shown in the legend. QLD clades and subclades are shaded and named.
(PNG)

## References

[1] Majowicz SE, Musto J, Scallan E, Angulo FJ, Kirk M, O'Brien SJ, et al The Global Burden of Nontyphoidal Salmonella Gastroenteritis. Clinical Infectious Diseases. 2010;50(6):882–9. doi: 10.1086/650733 20158401 10.1086/650733

[2] Ford L, Glass K, Veitch M, Wardell R, Polkinghorne B, Dobbins T, et al Increasing Incidence of Salmonella in Australia, 2000–2013. PLOS ONE. 2016;11(10):e0163989 doi: 10.1371/journal.pone.0163989 27732615 10.1371/journal.pone.0163989 PMC5061413

[3] Department of Health. Australian national notifiable diseases and case definitions [Internet]. Canberra (ACT): Commonwealth of Australia;; 2015 [cited 2017 07/09/2017]. Available from: http://www.health.gov.au/casedefinitions.

[4] Hendriksen RS, Vieira AR, Karlsmose S, Lo Fo Wong DMA, Jensen AB, Wegener HC, et al Global Monitoring of Salmonella Serovar Distribution from the World Health Organization Global Foodborne Infections Network Country Data Bank: Results of Quality Assured Laboratories from 2001 to 2007. Foodborne Pathogens and Disease. 2011;8(8):887–900. doi: 10.1089/fpd.2010.0787 21492021 10.1089/fpd.2010.0787

[5] OzFoodNet Working Group. Monitoring the incidence and causes of diseases potentially transmitted by food in Australia: annual report of the OzFoodNet Network, 2009. Communicable diseases intelligence quarterly report. 2010;34(4):396–426. Epub 2011/03/19. . 21413526 10.33321/cdi.2010.34.40

[6] OzFoodNet Working Group. Monitoring the incidence and causes of diseases potentially transmitted by food in Australia: Annual report of the OzFoodNet network, 2011. Communicable diseases intelligence quarterly report. 2015;39(2):E236–64. Epub 2015/08/04. . 26234259 10.33321/cdi.2015.39.22

[7] Allard MW, Luo Y, Strain E, Pettengill J, Timme R, Wang C, et al On the Evolutionary History, Population Genetics and Diversity among Isolates of *Salmonella* Enteritidis PFGE Pattern JEGX01.0004. PLoS ONE. 2013;8(1):e55254 doi: 10.1371/journal.pone.0055254 23383127 10.1371/journal.pone.0055254 PMC3559427

[8] Bolger AM, Lohse M, Usadel B. Trimmomatic: a flexible trimmer for Illumina sequence data. Bioinformatics (Oxford, England). 2014;30(15):2114–20. Epub 2014/04/04. doi: 10.1093/bioinformatics/btu170 ; PubMed Central PMCID: PMCPmc4103590. 24695404 10.1093/bioinformatics/btu170 PMC4103590

[9] Stamatakis A. RAxML-VI-HPC: maximum likelihood-based phylogenetic analyses with thousands of taxa and mixed models. Bioinformatics (Oxford, England). 2006;22(21):2688–90. doi: 10.1093/bioinformatics/btl446 16928733 10.1093/bioinformatics/btl446

[10] Bankevich A, Nurk S, Antipov D, Gurevich AA, Dvorkin M, Kulikov AS, et al SPAdes: a new genome assembly algorithm and its applications to single-cell sequencing. J Comput Biol. 2012;19(5):455–77. Epub 2012/04/18. doi: 10.1089/cmb.2012.0021 ; PubMed Central PMCID: PMCPmc3342519. 22506599 10.1089/cmb.2012.0021 PMC3342519

[11] Rissman AI, Mau B, Biehl BS, Darling AE, Glasner JD, Perna NT. Reordering contigs of draft genomes using the Mauve Aligner. Bioinformatics (Oxford, England). 2009;25(16):2071–3. doi: 10.1093/bioinformatics/btp356 19515959 10.1093/bioinformatics/btp356 PMC2723005

[12] Achtman M, Wain J, Weill F-X, Nair S, Zhou Z, Sangal V, et al Multilocus Sequence Typing as a Replacement for Serotyping in Salmonella enterica. PLOS Pathogens. 2012;8(6):e1002776 doi: 10.1371/journal.ppat.1002776 22737074 10.1371/journal.ppat.1002776 PMC3380943

[13] Zankari E, Hasman H, Cosentino S, Vestergaard M, Rasmussen S, Lund O, et al Identification of acquired antimicrobial resistance genes. The Journal of antimicrobial chemotherapy. 2012;67(11):2640–4. Epub 2012/07/12. doi: 10.1093/jac/dks261 ; PubMed Central PMCID: PMCPmc3468078. 22782487 10.1093/jac/dks261 PMC3468078

[14] Jia B, Raphenya AR, Alcock B, Waglechner N, Guo P, Tsang KK, et al CARD 2017: expansion and model-centric curation of the comprehensive antibiotic resistance database. Nucleic Acids Res. 2017;45(D1):D566–d73. Epub 2016/10/30. doi: 10.1093/nar/gkw1004 ; PubMed Central PMCID: PMCPmc5210516. 27789705 10.1093/nar/gkw1004 PMC5210516

[15] Zhang S, Yin Y, Jones MB, Zhang Z, Deatherage Kaiser BL, Dinsmore BA, et al Salmonella Serotype Determination Utilizing High-throughput Genome Sequencing Data. Journal of Clinical Microbiology. 2015 doi: 10.1128/jcm.00323-15 25762776 10.1128/JCM.00323-15 PMC4400759

[16] Marttinen P, Hanage WP, Croucher NJ, Connor TR, Harris SR, Bentley SD, et al Detection of recombination events in bacterial genomes from large population samples. Nucleic Acids Res. 2012;40(1):e6 Epub 2011/11/09. doi: 10.1093/nar/gkr928 ; PubMed Central PMCID: PMCPmc3245952. 22064866 10.1093/nar/gkr928 PMC3245952

[17] Porwollik S, Santiviago CA, Cheng P, Florea L, McClelland M. Differences in Gene Content between Salmonella enterica Serovar Enteritidis Isolates and Comparison to Closely Related Serovars Gallinarum and Dublin. Journal of Bacteriology. 2005;187(18):6545–55. doi: 10.1128/JB.187.18.6545-6555.2005 16159788 10.1128/JB.187.18.6545-6555.2005 PMC1236623

[18] Hanna LF, Matthews TD, Dinsdale EA, Hasty D, Edwards RA. Characterization of the ELPhiS Prophage from Salmonella enterica Serovar Enteritidis Strain LK5. Applied and Environmental Microbiology. 2012;78(6):1785–93. doi: 10.1128/AEM.07241-11 22247173 10.1128/AEM.07241-11 PMC3298174

[19] Kwag SI, Bae DH, Cho JK, Lee HS, Ku BG, Kim BH, et al Characteristics of persistent Salmonella Enteritidis strains in two integrated broiler chicken operations of Korea. The Journal of veterinary medical science. 2008;70(10):1031–5. Epub 2008/11/05. . 18981657 10.1292/jvms.70.1031

[20] Hendriksen RS, Hyytia-Trees E, Pulsrikarn C, Pornruangwong S, Chaichana P, Svendsen CA, et al Characterization of Salmonella enterica serovar Enteritidis isolates recovered from blood and stool specimens in Thailand. BMC Microbiology. 2012;12(1):92 doi: 10.1186/1471-2180-12-92 22672324 10.1186/1471-2180-12-92 PMC3583215

[21] Asai T, Harada K, Kojima A, Sameshima T, Takahashi T, Akiba M, et al Phage type and antimicrobial susceptibility of Salmonella enterica serovar Enteritidis from food-producing animals in Japan between 1976 and 2004. The new microbiologica. 2008;31(4):555–9. Epub 2009/01/07. . 19123313

[22] Anonymous. Salmonella enteritidis phage type 21b. Communicable disease report CDR weekly. 1996;6(13):109 Epub 1996/03/29. . 8881601

[23] Ashton PM, Nair S, Peters TM, Bale JA, Powell DG, Painset A, et al Identification of Salmonella for public health surveillance using whole genome sequencing. PeerJ. 2016;4:e1752 doi: 10.7717/peerj.1752 27069781 10.7717/peerj.1752 PMC4824889

[24] Feasey NA, Hadfield J, Keddy KH, Dallman TJ, Jacobs J, Deng X, et al Distinct Salmonella Enteritidis lineages associated with enterocolitis in high-income settings and invasive disease in low-income settings. Nat Genet. 2016;48(10):1211–7. doi: 10.1038/ng.3644 http://www.nature.com/ng/journal/vaop/ncurrent/abs/ng.3644.html#supplementary-information. PubMed Central PMCID: PMCPMC5047355. 27548315 10.1038/ng.3644 PMC5047355

[25] Zheng J, Pettengill J, Strain E, Allard MW, Ahmed R, Zhao S, et al Genetic Diversity and Evolution of Salmonella enterica Serovar Enteritidis Strains with Different Phage Types. Journal of Clinical Microbiology. 2014;52(5):1490–500. doi: 10.1128/JCM.00051-14 24574287 10.1128/JCM.00051-14 PMC3993623

[26] Deng X, Desai PT, den Bakker HC, Mikoleit M, Tolar B, Trees E, et al Genomic Epidemiology of Salmonella enterica Serotype Enteritidis based on Population Structure of Prevalent Lineages. Emerging Infectious Diseases. 2014;20(9):1481–9. doi: 10.3201/eid2009.131095 25147968 10.3201/eid2009.131095 PMC4178404

[27] Betancor L, Yim L, Fookes M, Martinez A, Thomson NR, Ivens A, et al Genomic and phenotypic variation in epidemic-spanning Salmonella enterica serovar Enteritidis isolates. BMC Microbiology. 2009;9(1):237 doi: 10.1186/1471-2180-9-237 19922635 10.1186/1471-2180-9-237 PMC2784474

[28] Thomson NR, Clayton DJ, Windhorst D, Vernikos G, Davidson S, Churcher C, et al Comparative genome analysis of Salmonella Enteritidis PT4 and Salmonella Gallinarum 287/91 provides insights into evolutionary and host adaptation pathways. Genome Research. 2008;18(10):1624–37. doi: 10.1101/gr.077404.108 18583645 10.1101/gr.077404.108 PMC2556274

[29] Vishwakarma V, Periaswamy B, Bhusan Pati N, Slack E, Hardt W-D, Suar M. A Novel Phage Element of Salmonella enterica Serovar Enteritidis P125109 Contributes to Accelerated Type III Secretion System 2-Dependent Early Inflammation Kinetics in a Mouse Colitis Model. Infection and Immunity. 2012;80(9):3236–46. doi: 10.1128/IAI.00180-12 22753379 10.1128/IAI.00180-12 PMC3418750

[30] Blondel CJ, Jiménez JC, Contreras I, Santiviago CA. Comparative genomic analysis uncovers 3 novel loci encoding type six secretion systems differentially distributed in Salmonella serotypes. BMC Genomics. 2009;10(1):354 doi: 10.1186/1471-2164-10-354 19653904 10.1186/1471-2164-10-354 PMC2907695

[31] Matthews TD, Schmieder R, Silva GGZ, Busch J, Cassman N, Dutilh BE, et al Genomic Comparison of the Closely-Related Salmonella enterica Serovars Enteritidis, Dublin and Gallinarum. PLOS ONE. 2015;10(6):e0126883 doi: 10.1371/journal.pone.0126883 26039056 10.1371/journal.pone.0126883 PMC4454671

[32] Agron PG, Walker RL, Kinde H, Sawyer SJ, Hayes DC, Wollard J, et al Identification by Subtractive Hybridization of Sequences Specific for Salmonella enterica Serovar Enteritidis. Applied and Environmental Microbiology. 2001;67(11):4984–91. doi: 10.1128/AEM.67.11.4984-4991.2001 11679316 10.1128/AEM.67.11.4984-4991.2001 PMC93261

[33] Santiviago CA, Blondel CJ, Quezada CP, Silva CA, Tobar PM, Porwollik S, et al Spontaneous Excision of the Salmonella enterica Serovar Enteritidis-Specific Defective Prophage-Like Element φSE14. Journal of Bacteriology. 2010;192(8):2246–54. doi: 10.1128/JB.00270-09 20172996 10.1128/JB.00270-09 PMC2849447

[34] Shah DH, Zhou X, Kim H-Y, Call DR, Guard J. Transposon Mutagenesis of Salmonella enterica Serovar Enteritidis Identifies Genes That Contribute to Invasiveness in Human and Chicken Cells and Survival in Egg Albumen. Infection and Immunity. 2012;80(12):4203–15. doi: 10.1128/IAI.00790-12 22988017 10.1128/IAI.00790-12 PMC3497420

[35] Chiu C-H, Tang P, Chu C, Hu S, Bao Q, Yu J, et al The genome sequence of Salmonella enterica serovar Choleraesuis, a highly invasive and resistant zoonotic pathogen. Nucleic Acids Research. 2005;33(5):1690–8. doi: 10.1093/nar/gki297 15781495 10.1093/nar/gki297 PMC1069006

[36] Fuentes JA, Villagra N, Castillo-Ruiz M, Mora GC. The Salmonella Typhi hlyE gene plays a role in invasion of cultured epithelial cells and its functional transfer to S. Typhimurium promotes deep organ infection in mice. Research in microbiology. 2008;159(4):279–87. Epub 2008/04/25. doi: 10.1016/j.resmic.2008.02.006 . 18434098 10.1016/j.resmic.2008.02.006

[37] McClelland M, Sanderson KE, Spieth J, Clifton SW, Latreille P, Courtney L, et al Complete genome sequence of Salmonella enterica serovar Typhimurium LT2. Nature. 2001;413(6858):852–6. http://www.nature.com/nature/journal/v413/n6858/suppinfo/413852a0_S1.html. doi: 10.1038/35101614 11677609 10.1038/35101614

[38] Parkhill J, Dougan G, James KD, Thomson NR, Pickard D, Wain J, et al Complete genome sequence of a multiple drug resistant Salmonella enterica serovar Typhi CT18. Nature. 2001;413(6858):848–52. Epub 2001/10/26. doi: 10.1038/35101607 . 11677608 10.1038/35101607

[39] Shah DH, Lee M-j, Park J-h, Lee J-h, Eo S-k, Kwon J-t, et al Identification of Salmonella gallinarum virulence genes in a chicken infection model using PCR-based signature-tagged mutagenesis. Microbiology. 2005;151(12):3957–68. doi: 10.1099/mic.0.28126–0 16339940 10.1099/mic.0.28126-0

[40] Vernikos GS, Parkhill J. Interpolated variable order motifs for identification of horizontally acquired DNA: revisiting the Salmonella pathogenicity islands. Bioinformatics (Oxford, England). 2006;22(18):2196–203. doi: 10.1093/bioinformatics/btl369 16837528 10.1093/bioinformatics/btl369

[41] Langridge GC, Fookes M, Connor TR, Feltwell T, Feasey N, Parsons BN, et al Patterns of genome evolution that have accompanied host adaptation in Salmonella. Proceedings of the National Academy of Sciences of the United States of America. 2015;112(3):863–8. doi: 10.1073/pnas.1416707112 25535353 10.1073/pnas.1416707112 PMC4311825

[42] Sabbagh SC, Forest CG, Lepage C, Leclerc J-M, Daigle F. So similar, yet so different: uncovering distinctive features in the genomes of Salmonella enterica serovars Typhimurium and Typhi. FEMS Microbiology Letters. 2010;305(1):1–13. doi: 10.1111/j.1574-6968.2010.01904.x 20146749 10.1111/j.1574-6968.2010.01904.x

[43] Magnet S, Courvalin P, Lambert T. Activation of the cryptic aac(6')-Iy aminoglycoside resistance gene of Salmonella by a chromosomal deletion generating a transcriptional fusion. J Bacteriol. 1999;181(21):6650–5. Epub 1999/11/05. ; PubMed Central PMCID: PMCPmc94128. 10542165 10.1128/jb.181.21.6650-6655.1999 PMC94128

[44] Thong KL, Ngoi ST, Chai LC, Teh CSJ. Quinolone Resistance Mechanisms Among Salmonella enterica in Malaysia. Microbial Drug Resistance. 2015;22(4):259–72. doi: 10.1089/mdr.2015.0158 26683630 10.1089/mdr.2015.0158

[45] Hur J, Kim JH, Park JH, Lee Y-J, Lee JH. Molecular and virulence characteristics of multi-drug resistant Salmonella Enteritidis strains isolated from poultry. The Veterinary Journal. 2011;189(3):306–11. doi: 10.1016/j.tvjl.2010.07.017 20822940 10.1016/j.tvjl.2010.07.017

[46] Zou M, Keelara S, Thakur S. Molecular Characterization of Salmonella enterica Serotype Enteritidis Isolates from Humans by Antimicrobial Resistance, Virulence Genes, and Pulsed-Field Gel Electrophoresis. Foodborne Pathogens and Disease. 2012;9(3):232–8. doi: 10.1089/fpd.2011.1012 22283616 10.1089/fpd.2011.1012

[47] Ben Salem R, Abbassi MS, García V, García-Fierro R, Fernández J, Kilani H, et al Antimicrobial drug resistance and genetic properties of Salmonella enterica serotype Enteritidis circulating in chicken farms in Tunisia. Journal of Infection and Public Health. 2017 http://dx.doi.org/10.1016/j.jiph.2017.01.012. 10.1016/j.jiph.2017.01.012 28215920

[48] Crump JA, Medalla FM, Joyce KW, Krueger AL, Hoekstra RM, Whichard JM, et al Antimicrobial resistance among invasive nontyphoidal Salmonella enterica isolates in the United States: National Antimicrobial Resistance Monitoring System, 1996 to 2007. Antimicrobial agents and chemotherapy. 2011;55(3):1148–54. Epub 2011/01/05. doi: 10.1128/AAC.01333-10 ; PubMed Central PMCID: PMCPmc3067073. 21199924 10.1128/AAC.01333-10 PMC3067073

[49] Carattoli A, Zankari E, Garcia-Fernandez A, Voldby Larsen M, Lund O, Villa L, et al In silico detection and typing of plasmids using PlasmidFinder and plasmid multilocus sequence typing. Antimicrobial agents and chemotherapy. 2014;58(7):3895–903. Epub 2014/04/30. doi: 10.1128/AAC.02412-14 ; PubMed Central PMCID: PMCPmc4068535. 24777092 10.1128/AAC.02412-14 PMC4068535

[50] Olson AB, Andrysiak AK, Tracz DM, Guard-Bouldin J, Demczuk W, Ng LK, et al Limited genetic diversity in Salmonella enterica serovar Enteritidis PT13. BMC Microbiol. 2007;7:87 Epub 2007/10/03. doi: 10.1186/1471-2180-7-87 ; PubMed Central PMCID: PMCPmc2137926. 17908316 10.1186/1471-2180-7-87 PMC2137926

[51] Zheng J, Keys CE, Zhao S, Meng J, Brown EW. Enhanced subtyping scheme for Salmonella enteritidis. Emerg Infect Dis. 2007;13(12):1932–5. Epub 2008/02/09. doi: 10.3201/eid1312.070185 ; PubMed Central PMCID: PMCPmc2876743. 18258051 10.3201/eid1312.070185 PMC2876743

[52] Abraham S, Groves MD, Trott DJ, Chapman TA, Turner B, Hornitzky M, et al Salmonella enterica isolated from infections in Australian livestock remain susceptible to critical antimicrobials. International Journal of Antimicrobial Agents. 2014;43(2):126–30. doi: 10.1016/j.ijantimicag.2013.10.014 24314826 10.1016/j.ijantimicag.2013.10.014

